# Spontaneous dissection limited to left gastric artery

**DOI:** 10.1002/ccr3.1120

**Published:** 2017-08-11

**Authors:** Masaki Tago, Yoshimasa Oda, Naoko E. Furukawa, Shu‐ichi Yamashita

**Affiliations:** ^1^ Department of General Medicine Saga University Hospital Japan

**Keywords:** Left gastric artery dissection, sudden epigastric pain

## Abstract

Spontaneous dissection without an aneurysm limited to left gastric artery is fairly rare. Physicians should be alert to the possibility of this condition in patients with sudden‐onset abdominal pain.

## Case

**Figure 1:**
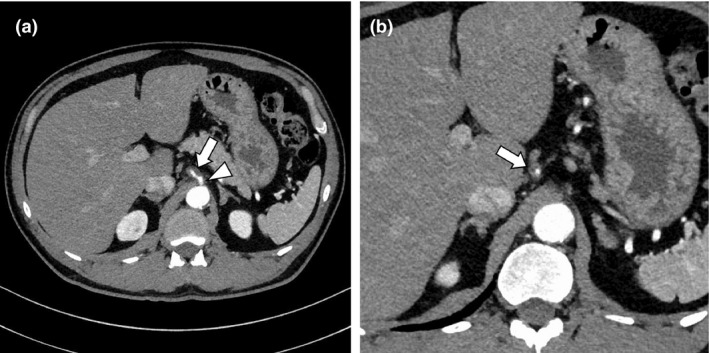
Abdominal contrast‐enhanced CT on admission. Low‐attenuated area was shown around the left gastric artery (a, b, arrows) arising from the abdominal aorta (a, arrowhead).

**Figure 2:**
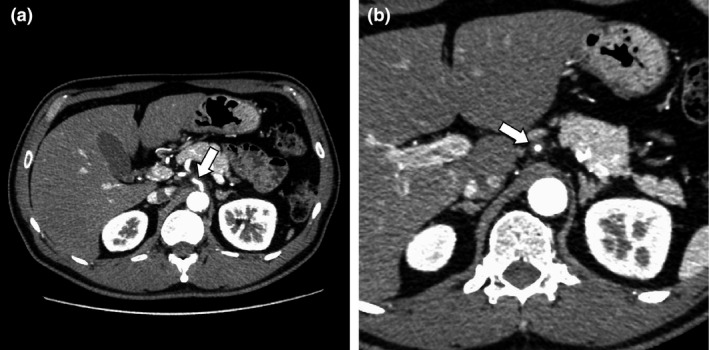
Follow‐up abdominal contrast‐enhanced CT 6 days later. The dilatation of true lumen of left gastric artery was shown in the follow‐up CT (a, b, arrows).

A 52‐year‐old man with history of hypertension developed sudden epigastric pain after dinner. Physical examination showed epigastric tenderness without guarding. His white blood cell count was 16,100 /*μ*L, and his C‐reactive protein concentration was 0.17 mg/dL. Abdominal contrast‐enhanced CT revealed a low‐attenuated area around left gastric artery (LGA) (Fig. 1a and b arrows) directly arising from the abdominal aorta (Fig. 1a arrowhead) without aneurysm formation or intestinal ischemia. We diagnosed spontaneous dissection without an aneurysm limited to LGA. Follow‐up CT 6 days later showed dilatation of true lumen (Fig. 2a and b arrows).

Spontaneous dissection without an aneurysm limited to LGA is fairly rare. Only four such cases have been reported as LGA dissection; only one was limited to LGA and involved no aneurysm 1. We found no cases involving the same anatomic variant of LGA as in our case, which occurs in 0.5–15.0% of the general population 2. There is not marked difference in severity between spontaneous dissection limited to LGA and that of celiac artery, but this anomaly requires precise knowledge of vascular anatomy.

Physicians should be alert to the possibility of this condition in patients with sudden‐onset abdominal pain.

## Authorship

MT: involved in literature search, concept, and drafting. YO: involved in clinical care of the patient. NEF: involved in drafting. SY: involved in concept and revision of article.

## Conflict of Interest

The authors state that they have no conflict of interest (COI).
